# Data-driven assessment of bioisosteric replacements and their influence on off-target activity profiles

**DOI:** 10.1039/d5md00686d

**Published:** 2025-11-06

**Authors:** Palle S. Helmke, Julia Kandler, Sara Ilie, Leo Gaskin, Gerhard F. Ecker

**Affiliations:** a University of Vienna, Department of Pharmaceutical Sciences Vienna Austria gerhard.f.ecker@univie.ac.at

## Abstract

Bioisosterism, a fundamental concept in medicinal chemistry, involves the substitution of chemical groups with structural analogs that preserve similar physicochemical properties while potentially modulating potency or toxicity. To systematically investigate shifts in pChEMBL values upon such substitutions, we developed a KNIME workflow that extracts and analyzes compound pairs featuring literature-curated common bioisosteric exchanges. The workflow retrieves pChEMBL values across 88 off-targets from ChEMBL and supports decision-making through pair-level quality metrics such as the document consistency ratio and assay context consistency ratio, which assess the consistency of the source data. Our analysis revealed that ester-to-secondary-amide replacements at the muscarinic acetylcholine receptor M2 (CHMR2) result in a significant mean decrease in pChEMBL of 1.26 across 14 compound pairs (*p* < 0.01). In contrast, phenyl-to-furanyl substitutions at the adenosine A2A receptor (ADORA2A) led to a mean increase in pChEMBL of 0.58 across 88 compound pairs (*p* < 0.01). Furthermore, a second KNIME workflow was developed to assess selectivity profiles by analyzing pChEMBL shifts at secondary targets. Among 66 compound pairs active at both ADORA2A and ADORA1, the mean change at ADORA1 was only +0.14 ± 0.52, indicating a selective potency increase at ADORA2A. This exemplifies a potential case of increased potency at an off-target associated with adverse effects, while maintaining activity at a pharmacologically desirable target. Conversely, furanyl-to-phenyl replacements may selectively reduce undesired potency at ADORA2A while preserving potency at ADORA1. This framework enables systematic, data-driven evaluation of potency shifts induced by bioisosteric replacements, aiding in the identification of substitutions associated with off-target potency increases or decreases during lead optimization. The workflow offers a semi-automated, reproducible approach that integrates bioisostere generation, activity mapping, and statistical assessment in a single platform, making it readily adaptable to other compound series and target panels. In addition, it evaluates whether activity at other known targets remains unchanged, thereby providing an assessment of selectivity of the replacements. The workflow can be applied to prioritize replacement strategies that reduce off-target risks, evaluate selectivity profiles, and generate curated potency shift data to support predictive modeling efforts.

## Abstract 2

Literature-curated classical and non-classical bioisosteric replacements from ChEMBL reveal their impact on off-target potency *via* a KNIME workflow.

## Graphical abstract

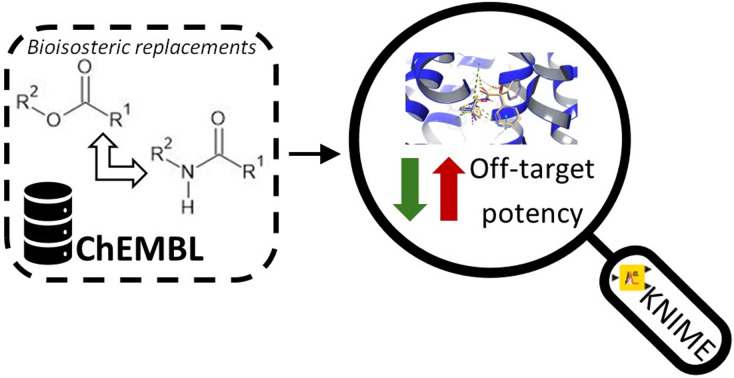

## 1 Introduction

Bioisosterism, rooted in the concept of isosterism, involves replacing one molecular fragment with another that retains similar steric or electronic characteristics.^1^ In medicinal chemistry, such replacements are widely employed to improve potency, selectivity, and pharmacokinetic profiles.^2^ Classical bioisosteres, such as –OH and –NH_2_, share valency and size, whereas non-classical bioisosteres mimic biological effects through spatial or electrostatic similarity.^1,3^ Beyond optimizing primary target interactions, these transformations can also modulate off-target binding and reduce toxicity.^4,5^ Systematic evaluation of defined bioisosteric replacements, such as esters-to-secondary-amides, across pharmacologically relevant proteins thus supports a more rational design of safer drugs.

Computational tools have become indispensable for the systematic identification and analysis of bioisosteric replacements, enabling medicinal chemists to explore structure–activity relationships and optimize molecular properties efficiently. Among these tools, matched molecular pair (MMP) analysis, originally introduced by Hussain and Rea,^6^ has become one of the most widely used approaches. It has been implemented in platforms such as mmpdb,^7^ the Matcher web application,^8^ and workflow based environments like KNIME^9^ using RDKit and Vernalis nodes.^10^ Several other resources leverage MMP analysis to extract and organize bioisosteric replacements: the SwissBioisostere database catalogs transformations and their impact on potency,^11^ the Base of Bioisosterically Exchangeable Replacements (BoBER) mines curated bioisosteric and scaffold hopping replacements from ChEMBL using MMP analysis and similarity calculations,^12^ and BioisoIdentifier extracts bioisosteric replacements from the Protein Data Bank (PDB) and clusters them using unsupervised machine learning.^13^ Bajorath and colleagues have made significant contributions to the advancement of MMP analog analysis. This includes matched molecular series, which consider broader sets of structurally related compounds, enabling the derivation of structure–activity relationship (SAR) rules and exploration of potency modifying changes across large compound sets.^14–17^ Ertl and coworkers have developed cheminformatics-based methods for systematically identifying bioisosteric and scaffold-hopping replacements, and for proposing new core structures in lead optimization.^18–20^ Collectively, these tools and methodologies underpin contemporary approaches for rational compound design and optimization.

Existing computational approaches often analyze bioisosteric replacements at scale, covering wide chemical and biological spaces. In contrast, our method applies a focused strategy by examining a predefined set of well-established medicinal chemistry transformations across a curated panel of safety-relevant off-target proteins, as defined by Brennan *et al.*^21^ This focus on off-target pharmacology is particularly important, because unintended protein interactions frequently cause adverse drug reactions and contribute to clinical failure. Within this context, our analysis captures not only large potency shifts but also more moderate yet consistent changes. Some bioisosteric replacements alter potency at one off-target protein while leaving activity unchanged at another known target, an effect newly captured by our KNIME workflow, providing deeper insights into selective modulation across off-targets. Another key feature of the workflow is the use of decision-making ratios, which systematically capture and contextualize these effects, enabling transparent evaluation of bioisosteric replacements across proteins. Notably, when only a small number of bioisosteric replacement pairs are available for an off-target protein, meaningful trends can still emerge. To our knowledge, such combined analysis of differential activity and decision-making ratios has not been reported in other data-driven studies.

To enable broader applicability, we developed a semi-automated, modular KNIME workflow. It streamlines the analysis, allows easy adaptation to other datasets and bioisosteric transformations, and facilitates the identification of replacements that may contribute to the design of safer compounds. By systematically linking bioisosteric substitutions to changes in potency and off-target profiles, and providing quantitative decision-making ratios, this workflow can help medicinal chemists prioritize modifications, optimize compound selectivity, and make data-driven decisions during early-stage drug design.

## 2 Results and discussion

### 2.1 Overview of compound selection and bioisosteric replacements for 88 off-targets

Notably, after applying filters for exact molecular weight (≤600 Da), exclusion of ^2^H-, ^3^H-, and ^11^C-labeled isotopes, and removal of tripeptides and larger peptides, most compounds matching *para*-phenylene were found in the ChEMBL database, as shown in Table 1. Among the panel of 88 off-targets, *para*-phenylene was also associated with the highest number of unique bioactive compounds, totaling 42 811, including 38 756 with inhibitory activity and 4055 with activation. Furthermore, phenyl exhibited the highest number of original-bioisosteric replacement pairs with a total of 5278 pairs, comprising 4862 with inhibitory activity and 416 with activation.

**Table 1:** Compounds from the ChEMBL database containing the specified functional groups, represented across 88 off-targets, along with the corresponding bioisosteric pairs

	Compounds in ChEMBL	Unique bioactive compounds (at 88 off-targets)		Bioisosteric pairs (at 88 off-targets)	
		Activation	Inhibition	Activation	Inhibition
Ester	187 417	774	5330	44	400
Secondary amide	596 202	3778	30 281	71	810
Carboxylic acid	193 693	759	4268	47	403
*Ortho*-Phenylene	189 149	1583	10 780	228	2223
*Meta*-Phenylene	228 400	1554	17 560	172	2558
*Para*-Phenylene	645 053	4055	38 756	229	3149
Phenyl	433 700	2935	26 440	416	4862

### 2.2 Compound-target pair statistics and off-target coverage

**Fig. 1:**
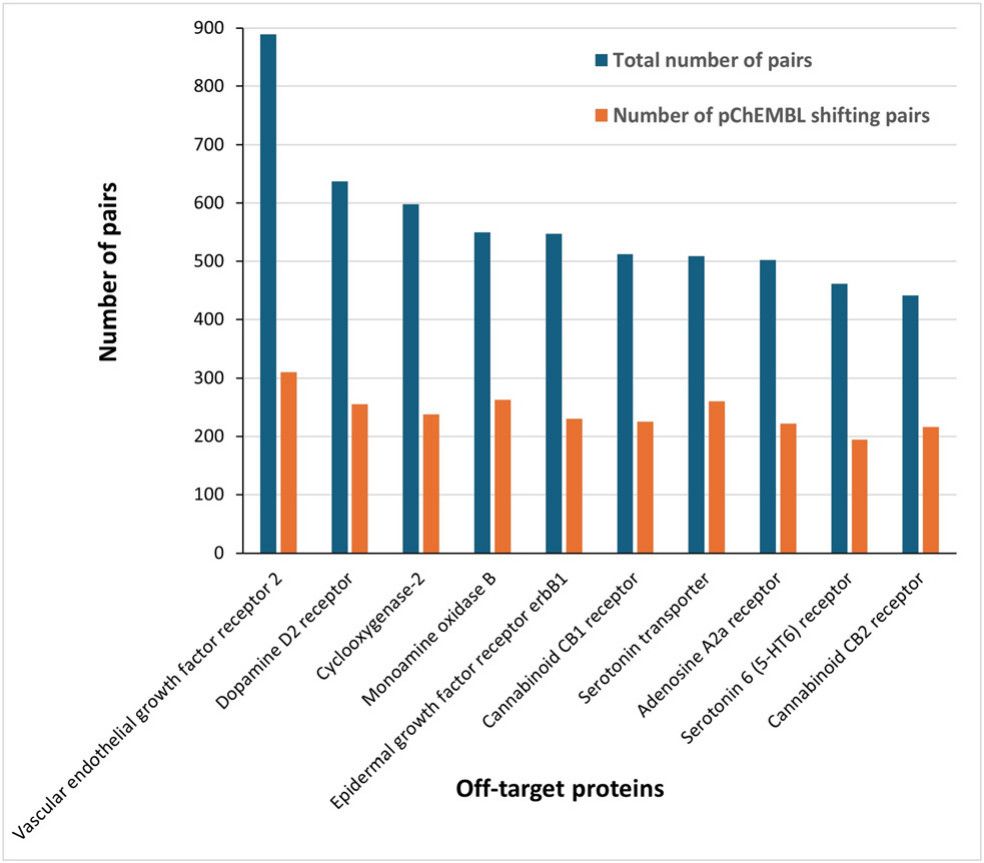
Top 10 off-targets ranked by frequency of bioisosteric replacement pairs and number of pairs with potency shifting substitutions (ΔpChEMBL ≥ 0.5).

Within the panel of 88 off-target proteins and the predefined replacement set, no bioisosteric compound pairs were identified for the Mas-related G-protein coupled receptor member X2, serine/threonine-protein kinase 35, and voltage-gated l-type calcium channel alpha1C subunit. As shown in Fig. 1, the vascular endothelial growth factor receptor 2 (VEGFR2) exhibited the highest number of bioisosteric replacement pairs and the most potency shifting substitutions (ΔpChEMBL ≥ 0.5). Among the top 10 off-targets with the highest number of bioisosteric replacement pairs, the distribution comprised five G protein-coupled receptors (GPCRs), two kinases, two enzymes of other classes, and one transporter. Notably, the human Ether-à-go-go-Related Gene (hERG) potassium channel, a common off-target protein known to cause preclinical and clinical safety failures due to its association with cardiotoxicity, particularly QT interval prolongation and risk of Torsade de Pointes, was ranked 12th.^22,23^

### 2.3 Assessment of bioisosteric effects on off-target potency

In the evaluated off-target panel and bioisosteric replacement space, 58 off-target replacement cases involving more than ten compound pairs exhibited statistically significant potency shifts (*p* < 0.1), with 56 of these reaching higher significance (*p* < 0.05). Among the 58 cases, 53 were associated with inhibition and five with activation. Significant cases included five cases for esters, six for secondary amides, four for carboxylic acids, 19 for phenyl, 12 for *ortho*-phenylene, nine for *meta*-phenylene, and three for *para*-phenylene. Importantly, all bioisosteric replacements can be interpreted in both directions. This means that ester-to-secondary-amide and secondary-amide-to-ester replacements were counted separately, even though they represent the same compound pairs evaluated in opposite directions.

### 2.4 Examples of bioisosteric shifts at selected off-target proteins

Three representative examples were selected for further analysis and discussion based on the relevance of the off-target protein, the number of available bioisosteric replacement pairs, and the magnitude of the observed mean change in bioactivity. These examples highlight cases where bioisosteric substitutions led to notable shifts in potency. The mean change in pChEMBL values and the associated significance reported in Table 2 were calculated as the average of the individual differences between each original-replacement compound pair.

**Table 2:** Impact of bioisosteric replacements on potency at off-target proteins, showing mean pChEMBL changes with pair counts, statistical significance (*p*-values) and decision-making ratios. (mean orig. pChEMBL = mean pChEMBL value of the original compounds; mean repl. pChEMBL = mean pChEMBL value of the replacement compounds; SD = standard deviation; ACCR = assay context consistency ratio; STCR = standard type consistency ratio; SFCR = salt form consistency ratio; DCR (unique docs.) = document consistency ratio based on unique sources)

Replacement	Target	Mean orig. pChEMBL	Mean repl. pChEMBL	Mean change of pChEMBL ± SD	Pair count	*p*-Value	Test	Mechanism of action	ACCR	STCR	SFCR	DCR (unique docs.)
Ester-to-secondary-amide	CHRM2	7.55	6.28	−1.26 ± 0.70	14	1.4 × 10^−5^	Paired *t*-test	Inhibition	0.93	0.93	1	0.50 (5)
Phenyl-to-cyclohexyl	ADORA2A	7.26	6.41	−0.86 ± 0.85	17	1.0 × 10^−3^	Paired *t*-test	Inhibition	0.94	1	1	0.94 (13)
Phenyl-to-furanyl	ADORA2A	7.07	7.65	+0.58 ± 0.87	88	1.3 × 10^−8^	Paired *t*-test	Inhibition	0.90	0.92	1	0.67 (52)

At the muscarinic acetylcholine receptor M2 (CHRM2), replacement of an ester with a secondary amide resulted in an approximate one order of magnitude decrease in activity across 14 compound pairs. Furthermore, substituting a phenyl with a cyclohexyl ring at the adenosine A2a receptor (ADORA2A) resulted in a mean pChEMBL decrease of 0.86 across 17 compound pairs. These substitutions are thus pointing towards a decrease in off-target potency. Also, at ADORA2A, replacing a phenyl with a furanyl group resulted in a mean pChEMBL increase of 0.58 across 88 compound pairs. Importantly, reversing these substitutions resulted in the opposite effects. Detailed statistics for all 58 cases are provided in the SI (Table S1). To support informed decision-making, four metrics were developed to assess the reliability of observed potency shifts: assay context (ACCR), standard type (STCR), salt form (SFCR), and document consistency (DCR) ratios. For example, in the phenyl-to-furanyl replacement set at ADORA2A, the assay context consistency ratio was 0.90, the standard type consistency ratio was 0.92, the salt form consistency ratio was 1.00, and the document consistency ratio was 0.67, based on data from 52 publications (Table 2). The observed potency increase is supported by high consistency in assay conditions, standard types, and compound forms, while some variability across sources (document consistency) informs the confidence level of this observation. Low consistency ratios or cases where all replacement pairs were measured in a single document suggest that potency changes should be interpreted with caution, as they may reflect experimental variability or study-specific biases, respectively, rather than true effects. A shift may be considered highly reliable if all consistency ratios equal 1 and the number of unique source documents matches the number of compound pairs, while the degree of confidence for other cases can be judged on these metrics.

### 2.5 Extended evaluation of potency shifts at an off-target while preserving activity at a known target

For all bioisosteric replacements with significant pChEMBL shifts, a second workflow was established to assess whether the same compound pairs also retained activity at another known target (*i.e.*, a target with experimentally reported interactions; non-shift target in Table 3). For example, the substitution of phenyl with furanyl at ADORA2A led to a mean pChEMBL increase of 0.58 across 88 compound pairs. Analysis of compounds from these groups at the Adenosine A1 receptor (ADORA1) revealed a smaller difference of 0.14 ± 0.52 in 66 compound pairs. A third exchange, replacing a phenyl with a cyclohexyl ring, yielded a mean pChEMBL decrease of 0.86, however within this group, a smaller difference of 0.21 ± 0.82 (statistically not significant due to high standard deviation) was observed at ADORA1 for 11 compound pairs. Notably, ADORA1 is not included in the 88 off-targets reported by Brennan *et al.*, representing a scenario in which potency at this target is maintained while off-target potency decreases. All 16 cases showing shifts at an off-target protein, accompanied by only minor changes at another known target (based on more than five compound pairs), are summarized in the SI (Table S2).

**Table 3:** Summary of bioisosteric exchanges with target-specific shifts in pChEMBL, showing minimal changes at other known targets. (mean orig. pChEMBL at non-shift target = mean pChEMBL value of the original compounds at the known non-shifting target; mean repl. pChEMBL = mean pChEMBL value of the replacement compounds at the known non-shifting target; SD = standard deviation)

Replacement	Shift target	Shift target mean change	Non-shift target	Mean orig. pChEMBL at non-shift target	Mean repl. pChEMBL at non-shift target	Pair count at non-shift target	Non-shift target mean change ± SD	Mechanism of action at non-shift target
Phenyl-to-cyclohexyl	ADORA2A	−0.86	ADORA1	6.93	6.73	11	−0.21 ± 0.82	Inhibition
Phenyl-to-furanyl	ADORA2A	+0.58	ADORA1	7.11	7.25	66	0.14 ± 0.52	Inhibition

### 2.6 Docking into muscarinic acetylcholine receptor M2

In 14 cases involving bioisosteric substitutions of esters with secondary amides at CHRM2, a decrease in potency was observed, with mean pChEMBL values dropping from 7.55 (0.028 μMolar, esters) to 6.28 (0.53 μMolar, secondary amides). To explore the structural basis of this effect, two representative compounds – CHEMBL558910 (ester), which was co-crystallized in CHRM2 (PDB ID: 3UON)^24^ and CHEMBL3401640 (amide) – were docked into the receptor using Maestro (Schrödinger Release 2022-4). CHRM2 was selected for this analysis because the availability of a co-crystallized ester ligand from the 14 pairs allowed redocking of the ester and docking of the corresponding amide, providing a rapid proof-of-concept validation of the workflow.

**Fig. 2:**
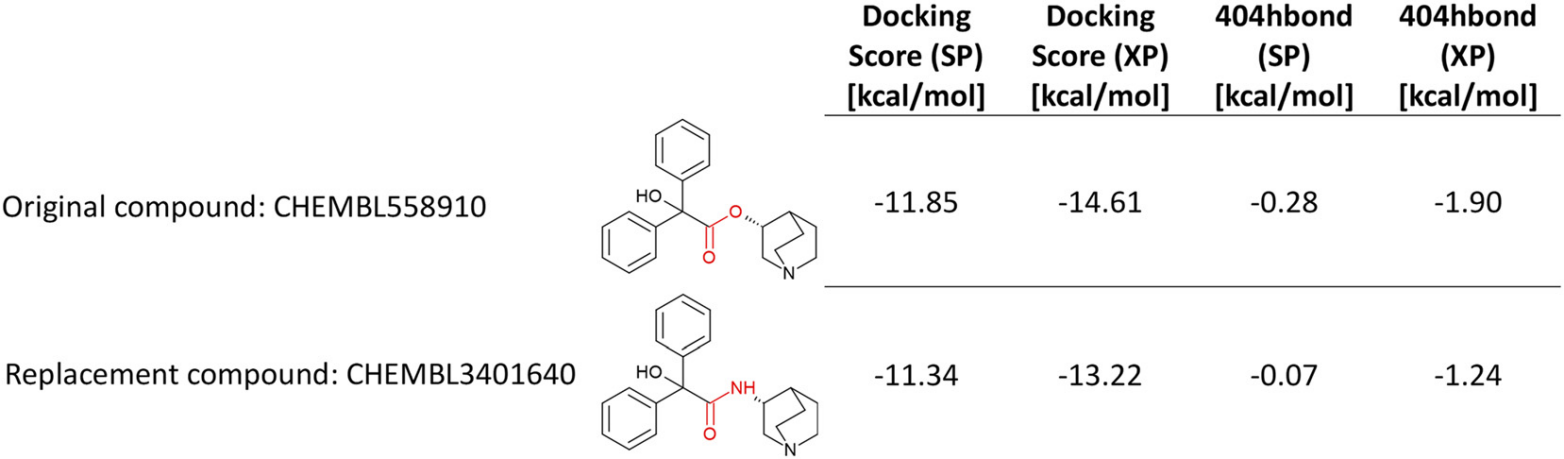
Docking scores for the same enantiomers of CHEMBL558910 (ester) and CHEMBL3401640 (secondary amide) at CHRM2, illustrating reduced binding affinity and weaker hydrogen bonding with Asn404 of the amide analog.

**Fig. 3:**
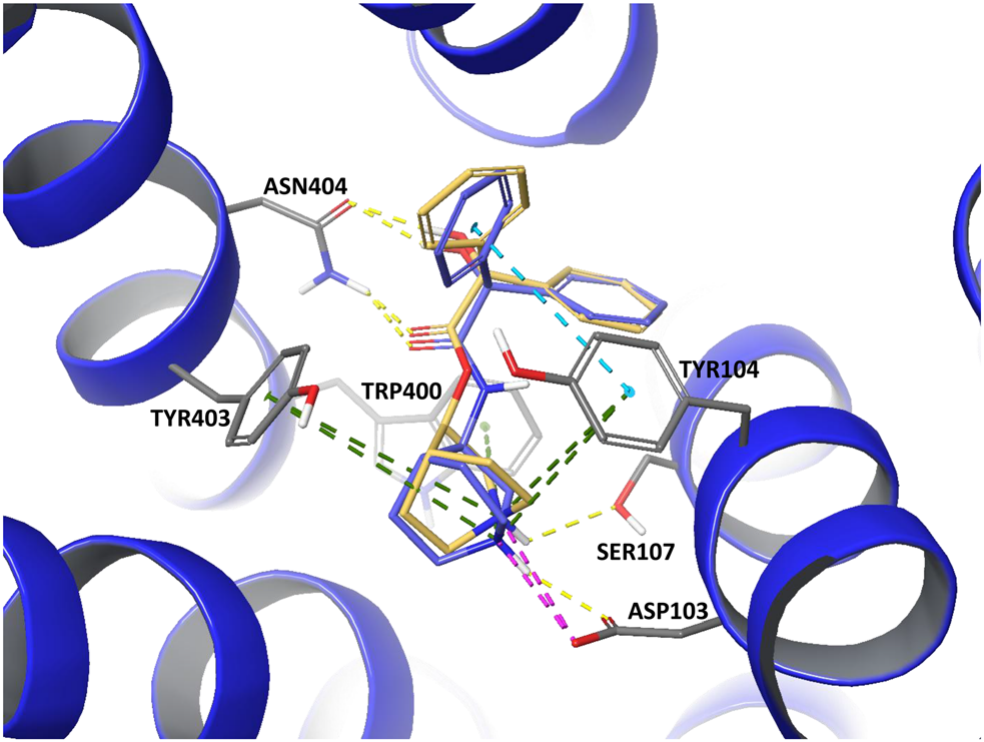
Docking poses of the ester (CHEMBL558910, yellow carbon atoms) and its corresponding amide (CHEMBL341640, purple carbon atoms) in CHRM2 (PDB ID: 3UON),^24^ generated using Maestro (Schrödinger Release 2022-4). Key residues involved in binding are shown with their interactions color-coded as follows: hydrogen bonds in yellow, salt bridges in pink, π–π stacking in turquoise, and π-cation interactions in green.

Fig. 2 summarizes the docking scores and hydrogen bonding interactions with Asn404 for the same enantiomers of the ester and its corresponding amide. The amide consistently exhibits higher (less favorable) docking scores and weaker hydrogen bonding with Asn404 compared to the ester. The corresponding docking poses and molecular interactions are illustrated in Fig. 3 (ester: carbon atoms shown in yellow, amide: carbon atoms shown in purple). Both compounds engage in a salt bridge with Asp103 and π-cation interactions with Tyr104, Trp400, and Tyr403, alongside hydrogen bonding with Asn404. In addition to these shared interactions, the amide forms a π–π stacking between one of its phenyl rings and Tyr104, as well as a hydrogen bond to the carbonyl oxygen of Asp103. Conversely, the ester's positively charged nitrogen establishes an additional hydrogen bond with Ser107.

In this study, we applied commonly used bioisosteric replacements from medicinal chemistry to systematically investigate their effects on a defined panel of 88 off-target proteins. This study aimed to assess whether consistent bioisosteric replacement trends in the ChEMBL dataset could help reduce off-target activity for specific targets across groups of compounds. To this end, we examined compound pairs differing only by a single bioisosteric substitution and evaluated off-target potency decreases or increases, with three representative cases illustrated. Of these, two exemplify a particularly informative scenario in which a bioisosteric exchange preserves potency at a known target while reducing potency at a secondary off-target. The observed potency shifts were further examined with respect to the underlying mechanistic rationale associated with the bioisosteric replacements, thereby illustrating the applicability of the KNIME workflow as a systematic approach to investigating such structure–activity relationships.

The muscarinic acetylcholine receptor M2 (CHRM2), part of the Gi/Go family of G protein-coupled receptors, is central to parasymphathetic regulation of cardiovascular function *via* activation of G protein-coupled inwardly-rectifying potassium (GIRK) channels.^24^ It is the only muscarinic acetylcholine receptor subtype known to directly modulate cardiac parameters such as heart rate and contractility.^25^ The receptor is widely expressed in the heart, brain, urinary bladder, and gastrointestinal tract, and has been extensively characterized pharmacologically, with both orthosteric and allosteric ligands.^24,26^ The physiological relevance of muscarinic receptors has made them important targets in the development of drugs for conditions such as Parkinson's and Alzheimer's disease.^27^ However, M2-selective antagonists are not widely used clinically due to poor selectivity and the risk of cardiac side effects, particularly tachycardia.^28^

Replacing an ester with a secondary amide at CHRM2 led in 14 cases to a potency decrease of slightly more than one order of magnitude in pChEMBL. Docking scores indicate higher binding affinity for the ester-containing compound (CHEMBL558910) compared to its amide analogue (CHEMBL341640), consistent with weaker hydrogen bonding of the amide to Asn404 (Fig. 3). It has been proposed by Haga *et al.* that Asn404^6.52^ forms a stabilizing hydrogen bond with the ester group of 3-quinuclidinyl-benzilate (CHEMBL558910).^24^ However, Korczynska *et al.* reported that switching from an ester to an amide had little effect on total antagonist binding. Nevertheless, more broadly, they observed that compounds lacking the ester R1-moiety lost binding cooperativity.^29^ The discrepancy with our results, which show a potency decrease of more than one order of magnitude upon ester-to-secondary amide substitution, can be explained by differences in compound coverage and experimental readouts: the triazolo-quinazoline analogs studied by Korczynska *et al.* were not part of the ChEMBL dataset analyzed here, and they reported allosteric EC50 values, whereas the observed potency decrease in our study refers specifically to inhibitory activity measured by IC_50_ and *K*_i_ values.

Similarly, Barlow *et al.* showed that replacing the ester with an amide in a series of diphenylacetic acid derivatives reduced affinity for muscarine-sensitive acetylcholine receptors in the guinea-pig ileum by 40- to 100-fold. With similar series of phenylacetic acid derivatives, the reduction had been only 2- to 4-fold. They suggested that the onium moiety bound the receptor in a similar manner in both esters and their corresponding amides, and that the difference in affinity primarily arose from reduced contributions of the phenyl groups in amides. According to their interpretation, this diminished binding may have resulted from the increased stiffness of the amide bond, which restricted the optimal positioning of the phenyl rings. This effect was more pronounced, in their analysis, in compounds that included two phenyl groups than in those with only one.^30^

At ADORA2A, replacing a phenyl group with a cyclohexyl moiety consistently reduced binding affinity by approximately one order of magnitude across 17 compound pairs. Structural studies indicate that the role of aromatic interactions at this site depends on the ligand binding mode. Jaakola *et al.* showed that the phenyl ring of the antagonist ZM241385 primarily forms hydrophobic interactions with Leu267^7.32^ and Met270^7.35^, suggesting that aromaticity may be less critical in this region.^31^ Supporting this, a ZM41385 derivative (LUF5477) with a cycloalkyl instead of a phenylmethylene group maintained high affinity, highlighting substituent flexibility in solvent-exposed-hydrophobic regions of the binding pocket.^31–33^ In contrast, co-crystal structures of other ADORA2A antagonists reveal key aromatic interactions involving the phenyl ring, such as π–π stacking with His250^6.52^ (T-shaped) and Trp246^6.48^ (stacked).^34^ Additionally, some ligands access an adjacent binding pocket stabilized by conformational changes in aromatic residues like Tyr9^1.35^ and Tyr271^7.36^.^33^ Substituting aromatic groups, such as methoxyphenyl, with aliphatic rings (*e.g.* cyclopropyl) disrupts these interactions and pocket engagements, often resulting in potency loss.^31,33,35^ These observations indicate that although some regions tolerate cycloalkyl groups, the variable position and orientation of the ADORA2A binding pocket can disrupt essential aromatic contacts upon phenyl-to-cyclohexyl substitution, resulting in pronounced, context-dependent losses of potency.^31^

In contrast to ADORA2A, where 17 compound pairs showed a significant potency shift upon substituting a phenyl with a cyclohexyl group, the same modification resulted in only a small change in potency in 11 pairs at the ADORA1 receptor. This observation is consistent with previous structural studies. Cheng *et al.* and Glukhova *et al.* identified a key structural variation at residue 270^7.35^: ADORA1 contains a threonine that allows access to a hydrophobic pocket, whereas the bulkier methionine in ADORA2A blocks it. This single residue variation functions as a “gatekeeper” and is thought to contribute to ligand selectivity between the subtypes.^36,37^ As a result, the hydrophobic pocket accommodating the cyclohexane moiety, observed in compounds like DU172 and other C-8 substituted ADORA1-selective antagonists, is accessible in ADORA1 but occluded in ADORA2A, limiting interactions with other key residues such as Met177^5.35^, Leu253^6.54^ and Thr257^6.57^.^36,38,39^

Another notable substitution involving ADORA2A was the replacement of a phenyl ring with a furanyl moiety, which resulted in an average increase in pChEMBL of approximately 0.6 across 88 compound pairs (Table 2). This observation suggests a potential potency increase through the furanyl moiety. Bolteau *et al.* reported an alternative binding mode in which the aromatic furanyl ring, positioned at the C4 of their compound, is oriented toward the bottom of the binding pocket. This configuration allows the oxygen atom of the furan ring to form a hydrogen bond with Asn253, thereby strengthening the interaction.^35,40^

Glukhova *et al.* showed that in their ADORA1 model, the residue Asn254^6.55^ (equivalent to Asn253^6.55^ in ADORA2A) interacts with the 6-oxy and N7 atoms of the xanthine-based ligand DU172, whereas in ADORA2A, the corresponding Asn253^6.55^ forms a hydrogen bond with the oxygen of the furan ring in ZM241385. This difference in hydrogen bonding pattern repositions DU172 deeper in the ADORA1 orthosteric site and is further stabilized by an interaction with Tyr12^1.35^, as well as multiple hydrophobic interactions.^36^ Therefore, a different ligand orientation within the ADORA1 binding pocket likely contributes to the smaller effect of furanyl substitution, as this pocket does not support the same interactions as ADORA2A, consistent with the modest average pChEMBL difference of 0.14 observed for the corresponding 66 compound pairs.

Substituting a phenyl with a furanyl moiety led to increased potency at ADORA2A, while the reversed substitution led to a decrease. This may mitigate ADORA2A-mediated side effects such as elevated blood pressure, increased heart rate, platelet aggregation, and aggression as reported by Lynch *et al.*^41^ This effect is relevant given the widespread expression of ADORA2A in the striatum, immune cells, spleen, thymus, blood platelets, heart, lung, and blood vessels, where it regulates inflammation, vascular tone, and neurotransmission.^42–46^ Importantly, this replacement preserves antagonistic activity at ADORA1, enabling diuretic effects useful in the treatment of fluid-retention disorders including congestive heart failure.^42,47^ In contrast, ADORA2A, when considered the intended target, benefits from enhanced antagonism upon replacement of a phenyl with a furanyl group. In the context of Parkinson's disease, ADORA2A antagonists slow down dopaminergic neurodegeneration, and demonstrate antidepressant properties.^47–49^ Replacing a phenyl with a cyclohexyl group similarly reduced activity at ADORA2A while retaining activity at ADORA1.

Trends in potency shifts across sets of bioisosteric replacements were analyzed using *p*-values to assess statistical significance. To support confident decision-making, four additional metrics were calculated. The assay context consistency ratio reflects how often replacements were tested under the same BioAssay Ontology (BAO) label, which organizes and standardizes high-throughput (HTS) assay data to improve comparability and interpretation.^50^ The salt form consistency ratio shows how often the same salt form or parent compound (non-salt) was used within the bioisosteric compound pairs, providing a sense of formulation-related variability. While salt forms do not typically alter the intrinsic potency at the molecular target, they can influence solubility, stability, or bioavailability.^51–53^ The standard type consistency ratio captures the proportion of replacements reported using the same activity type (*e.g.*, IC_50_*vs.* IC_50_), ensuring comparability of potency values. This is essential as IC_50_ and *K*_i_ may not be directly comparable without accounting for assay conditions and inhibition mechanisms.^54^ Notably, combining IC_50_ and *K*_i_ data from multiple sources can introduce significant noise, as demonstrated by Landrum and Riniker.^55^ To address this, the document consistency ratio captures how often data points for bioisosteric replacement pairs originate from the same publication. This metric helps distinguish between inter-assay variability, differences arising from experiments conducted under varying conditions in different laboratories, and more systematic effects. In contrast, when potency changes are mainly reported from a single publication or laboratory, intralaboratory variability or lab-specific biases may be sources of uncertainty.^56^ To assess such variability, it is useful to examine how consistently these effects are reported across studies. A higher number of unique documents supporting a given replacement may increase confidence in the observation and improve the generalizability of these findings, while also increasing inter-assay variability. Together, the four described metrics provide critical context on the consistency, comparability, and scope of the underlying data, enabling decision-makers to better judge the reliability of observed potency shifts.

## 3 Conclusion

We developed a semi-automated KNIME workflow to analyze frequently used classical and non-classical literature-curated bioisosteric replacements across a panel of 88 off-targets. By identifying subtle yet significant potency shifts, we uncovered 58 cases in which bioisosteric replacement pairs led to either increased or decreased off-target potency (ΔpChEMBL ≥ 0.5). Additional cases showed significant potency changes at an off-target without corresponding shifts at another known target. These findings provide a valuable resource for guiding safer bioisosteric design by highlighting sometimes nuanced off-target effects that might otherwise be overlooked. To support robust decision-making, we provide four metrics – assay context, standard type, salt form, and document consistency ratios between bioisosteric compound pairs, along with the number of unique source documents. Limitations of this study include the limited number of compound pairs available, which restricts the statistical power of the analysis. Moreover, only ChEMBL was used as a data source, and validation with additional databases would strengthen the findings. Furthermore, it remains uncertain whether the annotated off-target interactions truly represent off-targets for each compound. Finally, the observed trends in potency do not necessarily translate into differences in absorption, distribution, metabolism, excretion, or toxicity (ADMET) profiles, nor can it be concluded that they would lead to reduction in side effects, so the clinical relevance of the findings remains uncertain. Future work could explore its application to larger compound collections and integration with pharmacokinetic and safety profiling to further support medicinal chemistry efforts. In addition, coupling the workflow with data imputation and regression modeling approaches could help to fill in missing bioactivity values and better capture quantitative potency differences, thereby amplifying the effect size and reliability of reported changes between bioisosteric pairs. Ultimately, these computational extensions should be complemented by prospective experimental validation, providing direct evidence of whether the observed increases or decreases in potency translate into meaningful differences in biological systems and, ultimately, clinical outcomes. Beyond this, our analysis provides medicinal chemists with guidance in early drug discovery, enabling rapid analysis of their datasets to prioritize compound modifications to improve potency and reduce off-target effects.

## 4 Materials and methods

### 4.1 KNIME

KNIME (Konstanz Information Miner) Analytics Platform (version 4.7.7) was used for data processing, conducting bioisosteric replacements, and analysis. This open-source software provides a robust environment for data science through an intuitive, node-based interface.^9^

### 4.2 Data collection

Bioactivity data were retrieved from ChEMBL version 34.^57^ The dataset was filtered to include only bioactivity measurements for human targets with a standard relation of ‘=’. To ensure a precise distinction between different modes of action and their potential implications for side effects and toxicity, either inhibitory data (IC_50_, *K*_i_) or activation data (EC50) were selected. For compounds with multiple pChEMBL entries for a given target, the maximum pChEMBL value was selected within the defined mode of action to reflect the strongest reported potency. This choice may bias results toward higher apparent potency, while using the mean or median could underestimate high-potency interactions or be influenced by outliers. The maximum was chosen to ensure that the strongest observed potency, which is the most relevant for assessing potential off-target effects, is captured. Tripeptides and larger peptides were excluded, and only compounds with molecular weight equal or below 600 Da were retained to align with drug-like chemical space. Salts and charged forms were included as reported in ChEMBL. Compounds labeled with stable or radioactive isotopes such as deuterium (^2^H), tritium (^3^H), or carbon-11 (^11^C) were excluded from the analysis.

### 4.3 Functional group identification

Compounds containing predefined functional groups were identified using the RDKit^58^ substructure filter node in KNIME. SMARTS patterns were used to filter acyclic esters, secondary amides, carboxylic acids, as well as phenyl and phenylene groups as shown in Table 4. Cyclic esters (lactones) and amides (lactams) were excluded from the analysis. The list of functional groups can be extended as required.

**Table 4:** Substructures and their corresponding query SMARTS patterns used for substructure filtering

Substructure	Query SMARTS for substructure filter
Ester	[c,C:0]–[C;!r](=O)–O–[c,C:1]
Secondary amide	[c,C:0]–[C;!r](=O)–[NH;!r]–[c,C!$(C(=O))]
Carboxylic acid	[*:0]–C(=O)–[OH]
Phenyl	c1(:[cH1]:[cH1]:[cH1]:[cH1]:[cH1]:1)[*:1]
*Ortho*-Phenylene	[c;R1]1([*:1]):[c;R1]([*:0]):[cH1;R1]:[cH1;R1]:[cH1;R1]:[cH1;R1]:1
*Meta*-Phenylene	c1(:[cH1]:c(:[cH1]:[cH1]:[cH1]:1)–[*:1])–[*:0]
*Para*-Phenylene	c1(:[cH1]:[cH1]:c(:[cH1]:[cH1]:1)–[*:1])–[*:0]

### 4.4 Safety-relevant off-target panel

Only compounds containing the predefined substructures and reported bioactivity against at least one of the 88 human off-targets from Brennan *et al.* were retained for generating bioisosteric replacements. This curated panel includes pharmacologically important proteins frequently associated with adverse drug reactions, such as GPCRs, ion channels, and nuclear receptors.^21^ The list of targets can be adapted as needed to suit different toxicological profiling objectives.

### 4.5 Defining SMARTS reaction patterns (SMIRKS)

**Fig. 4:**
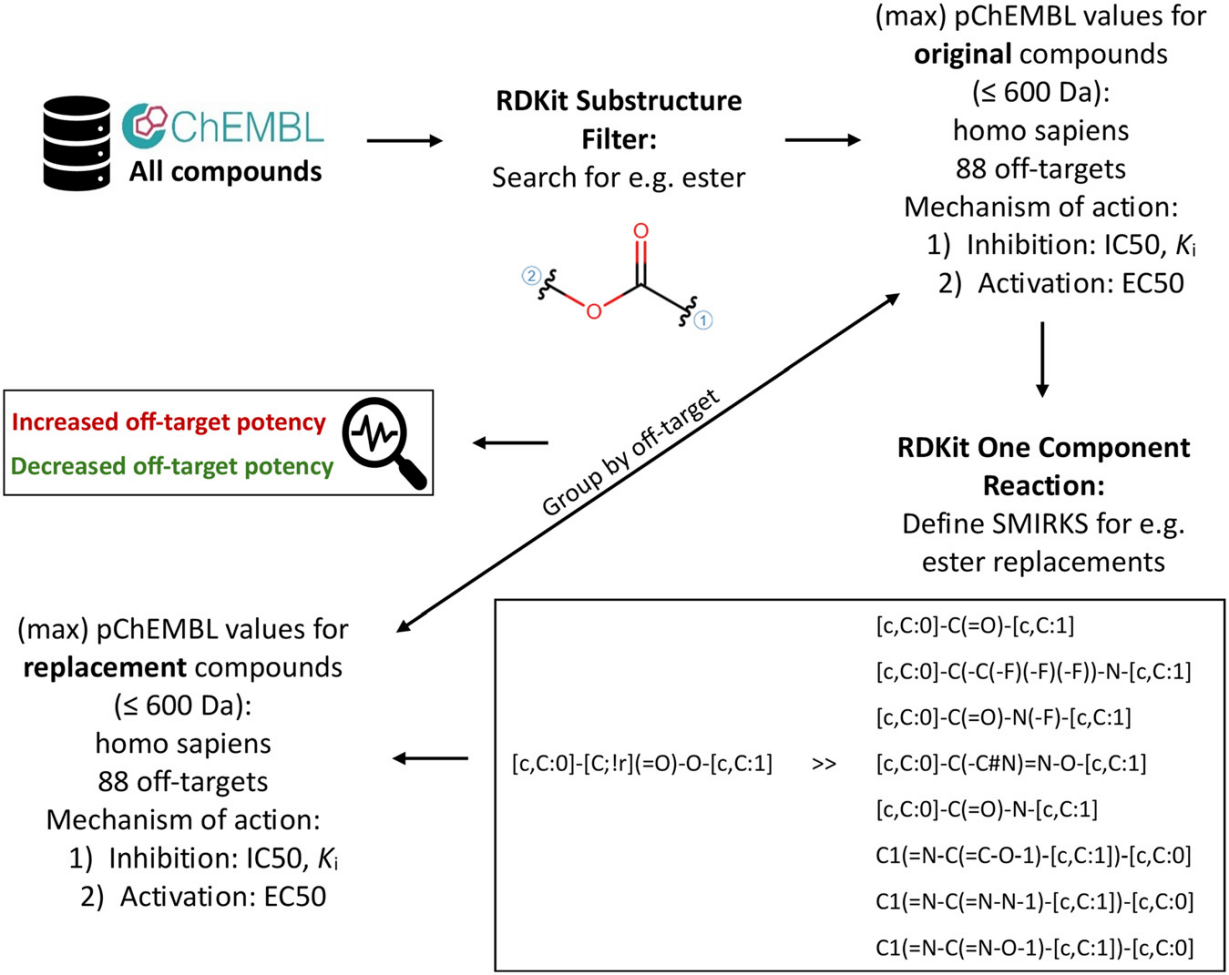
Workflow overview illustrating substructure retrieval, bioactivity annotation from ChEMBL, application of predefined bioisosteric replacement SMIRKS, and analysis of ΔpChEMBL values per off-target and per replacement to assess increases and decreases in off-target potency.

Bioisosteric replacements were generated using the RDKit one component reaction node in KNIME. For this purpose, eight SMIRKS patterns were defined for esters, five for secondary amides, seven for carboxylic acids, six for phenyl and 17 for phenylene groups: seven, six, and four for *ortho*-, *meta*-, and *para*-phenylene groups, respectively, as depicted in Fig. 4. SMIRKS (SMILES reaction extensions) encode generic reaction transformations using a text-based notation.^59^ These patterns were curated from three sources: the Cambridge MedChem Consulting bioisostere database,^60^ Jayashree *et al.*,^3^ and Stepan *et al.*^61^ To identify and correct false replacements, molecular weight differences between original and substituted compounds were calculated using RDKit descriptor nodes, as the expected weight difference for a given replacement type (*e.g.* ester-to-secondary-amide) is fixed between each original and replacement compound. The list of defined SMIRKS can be extended and adapted depending on the specific research focus.

### 4.6 Compound identifier retrieval and off-target activity mapping

Following bioisosteric replacements, the chemical structures of the new compounds were converted to InChI keys using the RDKit to InChI node. These InChI keys were mapped to ChEMBL IDs and subsequently linked to bioactivity data specifically reported for the panel of 88 off-target proteins (Fig. 4). If InChI keys were incorrectly generated, the corresponding InChI codes were used instead. Compound pairs consisting of an original molecule (*e.g.*, ester) and its bioisosteric replacement (*e.g.*, secondary amide) were identified based on the availability of reported bioactivity values for both compounds against the same off-target. Compound pairs were discarded when InChi key or InChi code information was missing for either the original or the replacement in ChEMBL. If both the parent (non-salt) and salt form pairs were present for a given off-target replacement combination, only the non-salt form pair was retained. All compound pairs representing the same type of bioisosteric replacement were then grouped by off-target, for example, all ester-to-secondary-amide pairs were grouped for CHRM2.

### 4.7 Statistical analysis

For each compound pair within the same replacement type and off-target protein, the difference in pChEMBL values between the original and replacement compound was separately calculated. Afterwards, these pairwise differences were averaged to obtain a mean shift in potency for each bioisosteric replacement type and off-target protein. A Shapiro–Wilk test assessed the normality of the distribution of these differences. If normality was confirmed, a paired *t*-test was applied; otherwise, the non-parametric Wilcoxon signed-rank test was used. Only combinations with more than 10 compound pairs, a statistically significant result (*p* < 0.1), and mean pChEMBL shifts larger than 0.5 were considered for further interpretation. A significance threshold of *p* < 0.1 was chosen to allow detection of subtle potency shifts in small compound pair sets. This relatively lenient threshold provides sensitivity for exploratory analysis while maintaining a minimum standard for statistical confidence. For more stringent evaluation, a stricter threshold of *p* < 0.05 was also applied.

### 4.8 Decision-making ratios

To ensure confident interpretation of bioactivity changes following bioisosteric replacements, four additional ratios were calculated per replacement type-off-target combination. First, the assay context consistency ratio assessed whether both the original and replacement compounds were tested under the same BioAssay Ontology (BAO) label (*e.g.*, cell-based *vs.* cell-based), which reflects the experimental format. Second, the standard type consistency ratio evaluated whether compounds were measured using the same bioactivity endpoint (*e.g.*, IC_50_*vs.* IC_50_). Third, the salt form consistency ratio determined if both compounds were either in salt form or non-salt form. Fourth, the document consistency ratio was calculated, indicating the proportion of bioisosteric replacement pairs whose bioactivity values originated from the same document. Additionally, the total number of unique documents per replacement and off-target protein was determined. All documents reporting either the original or replacement compound with a pChEMBL value were considered when counting unique documents per replacement type-off-target combination. Importantly, to isolate the effect of the structural replacement itself, only compound pairs with matching stereochemistry at all chiral centers were included in the analysis.

### 4.9 Extended evaluation of potency shifts at an off-target while preserving activity at a known target

To evaluate changes in off-target potency associated with bioisosteric replacements, we analyzed shifts in pChEMBL values between sets of compound-bioisostere pairs. There are increases and decreases in off-target potency associated with bioisosteric replacements. To further support this analysis, the KNIME workflow was extended to assess the number of compound pairs within each replacement-off-target combination that showed increased or decreased activity at an off-target but maintained potency at a known target.

### 4.10 Docking into M2 muscarinic acetylcholine receptor

To support the analysis of a significant off-target potency decrease involving the substitution of an ester with a secondary amide at CHRM2, a structure-based analysis was conducted. The docking protocol involved extracting the ligand from the crystal structure of CHRM2 (PDB ID: 3UON),^24^ duplicating it, and modifying one copy by replacing the ester group with a secondary amide. Both ligands were prepared using LigPrep (Schrödinger Release 2022-4: LigPrep, Schrödinger, LLC, New York, NY, 2022.) with default settings, except for the ionization, where the pH was set to 7.4 ± 0.4. Chirality was determined from the 3D structure. The receptor (PDB ID: 3UON) was prepared using the protein preparation (Schrödinger Release 2022-4: protein preparation workflow; Epik, Schrödinger, LLC New York, NY, 2022; Impact, Schrödinger, LLC, New York, NY; Prime, Schrödinger, LLC, New York, NY, 2022.) with default settings. The receptor grid was centered around the co-crystallized ligand and the grid size was set to “Dock ligands similar in size to the workspace ligand”. Docking was performed in Glide^62,63^ (Schrödinger Release 2022-4: Glide, Schrödinger, LLC New York, NY, 2022.) first in standard precision (SP) and subsequently in extra precision (XP) mode, both with default parameters. In contrast to SP, XP utilizes a different scoring function and conformational sampling protocol, which is computationally more expensive and assigns higher penalties on poor conformational complementarity between the ligand and the protein.^64^ The OPLS4 (ref. 65) forcefield was used in all preparation and docking steps.

## Author contributions

Palle S. Helmke conducted the study and wrote the manuscript. Julia Kandler performed the docking studies and wrote the corresponding sections. Sara Ilie and Leo Gaskin assisted with computational work. Gerhard F. Ecker conceptualized and supervised the project. All authors discussed the results and contributed to the final manuscript.

## Conflicts of interest

The authors declare no conflicts of interest.

## Supplementary Material

MD-016-D5MD00686D-s001

## Data Availability

The datasets and the KNIME workflow are available at PHAIDRA at https://phaidra.univie.ac.at/detail/o:2163182. Supplementary information (SI) providing a summary of significant pChEMBL increase and decrease cases is available. See DOI: https://doi.org/10.1039/d5md00686d.
