# Supplementary material for: Data-driven assessment of bioisosteric replacements and their influence on off-target activity profiles
Source: RSC Med Chem. 2025 Nov 6;16(12):6048–58. doi: 10.1039/d5md00686d (PMC12591391; doi:10.1039/d5md00686d)
Supplement: MD-016-D5MD00686D-s001 [file MD-016-D5MD00686D-s001.pdf]

Supplementary Information (SI) for RSC Medicinal Chemistry.
This journal is © The Royal Society of Chemistry 2025

Supporting Information

Data-driven assessment of bioisosteric replacements and their influence on off-target activity profiles

Palle S. Helmke, Julia Kandler, Sara Ilie, Leo Gaskin, Gerhard F. Ecker*

University of Vienna, Department of Pharmaceutical Sciences, Vienna, Austria

*Corresponding author email: gerhard.f.ecker@univie.ac.at

Table S1. Summary of 58 significant pChEMBL increase and decrease cases from bioisosteric replacements across 88 off-target proteins.

replacement_kind	Target ChEMBL ID	Mean original pChEMBL	Mean replacement pChEMBL	Mean change of pChEMBL ± standard deviation	p-value	Pair count	Test	Unique Documents	Document consistency ratio	Salt form consistency ratio	Assay context consistency ratio	Standard type consistency ratio	Mechanism of action
ester-to-secondary-amide	CHEMBL211	7.55	6.28	-1.26 ± 0.70	1.4×10^{-5}	14	paired t-test	5	0.50	1.00	0.93	0.93	Inhibition
ester-to-secondary-amide	CHEMBL216	7.65	6.62	-1.03 ± 0.62	3.1×10^{-5}	14	paired t-test	6	0.50	1.00	0.93	0.93	Inhibition
ester-to-secondary-amide	CHEMBL245	7.48	6.76	-0.72 ± 0.79	2.6×10^{-3}	16	paired t-test	6	0.44	0.88	0.94	0.94	Inhibition
ester-to-secondary-amide	CHEMBL252	6.23	7.29	+1.07 ± 0.82	2.6×10^{-3}	10	paired t-test	4	0.30	1.00	1.00	1.00	Inhibition
secondary-amide-to-ester	CHEMBL211	6.28	7.55	+1.26 ± 0.70	1.4×10^{-5}	14	paired t-test	5	0.50	1.00	0.93	0.93	Inhibition
secondary-amide-to-ester	CHEMBL216	6.62	7.65	+1.03 ± 0.62	3.1×10^{-5}	14	paired t-test	6	0.50	1.00	0.93	0.93	Inhibition
secondary-amide-to-ester	CHEMBL245	6.76	7.48	+0.72 ± 0.79	2.6×10^{-3}	16	paired t-test	6	0.44	0.88	0.94	0.94	Inhibition
secondary-amide-to-ester	CHEMBL252	7.29	6.23	-1.07 ± 0.82	2.6×10^{-3}	10	paired t-test	4	0.30	1.00	1.00	1.00	Inhibition
secondary-amide-to-secondary-sulfonamide	CHEMBL203	8.59	7.50	-1.10 ± 1.30	1.4×10^{-2}	12	paired t-test	15	0.58	1.00	0.83	1.00	Inhibition
COOH-to-primary-amide	CHEMBL233	6.33	6.89	+0.55 ± 0.95	1.1×10^{-2}	16	Wilcoxon Signed-Rank	7	1.00	1.00	1.00	1.00	Inhibition
COOH-to-primary-amide	CHEMBL236	5.96	6.59	+0.63 ± 1.10	3.1×10^{-2}	16	Wilcoxon Signed-Rank	14	0.81	1.00	0.88	0.94	Inhibition

replacement_kind	Target ChEMBL ID	Mean original pChEMBL	Mean replacement pChEMBL	Mean change of pChEMBL ± standard deviation	p-value	Pair count	Test	Unique Documents	Document consistency ratio	Salt form consistency ratio	Assay context consistency ratio	Standard type consistency ratio	Mechanism of action
COOH-to-primary-amide	CHEMBL237	7.35	8.32	+0.97 ± 0.60	3.9×10^{-7}	21	paired t-test	7	1.00	1.00	1.00	1.00	Inhibition
phenyl-to-cyclohexyl	CHEMBL251	7.26	6.41	-0.86 ± 0.85	1.0×10^{-3}	17	paired t-test	13	0.94	1.00	0.94	1.00	Inhibition
phenyl-to-cyclohexyl	CHEMBL298	8.32	8.86	+0.54 ± 0.90	2.1×10^{-2}	18	paired t-test	10	0.78	1.00	0.78	1.00	Inhibition
phenyl-to-furanyl	CHEMBL251	7.07	7.65	+0.58 ± 0.87	1.3×10^{-8}	88	paired t-test	52	0.67	1.00	0.90	0.92	Inhibition
phenyl-to-phenyl-and-meta-chlorine	CHEMBL203	6.68	7.24	+0.56 ± 0.91	8.8×10^{-4}	35	paired t-test	29	0.83	1.00	0.86	0.97	Inhibition
phenyl-to-phenyl-and-para-chlorine	CHEMBL240	5.55	6.22	+0.66 ± 0.77	2.6×10^{-5}	31	Wilcoxon Signed-Rank	26	0.87	1.00	0.90	0.97	Inhibition
phenyl-to-phenyl-and-para-chlorine	CHEMBL2815	6.63	7.22	+0.59 ± 0.56	9.1×10^{-3}	10	paired t-test	7	1.00	1.00	1.00	0.90	Inhibition
phenyl-to-phenyl-and-para-chlorine	CHEMBL3371	7.84	7.32	-0.52 ± 0.83	1.7×10^{-5}	56	paired t-test	42	0.80	0.98	0.95	0.89	Inhibition
phenyl-to-3-pyridinyl	CHEMBL203	6.90	6.30	-0.60 ± 0.66	1.4×10^{-3}	18	paired t-test	18	0.89	1.00	1.00	1.00	Inhibition
phenyl-to-3-pyridinyl	CHEMBL218	7.42	6.86	-0.56 ± 0.74	4.0×10^{-2}	10	paired t-test	14	0.60	1.00	0.70	0.90	Inhibition
phenyl-to-3-pyridinyl	CHEMBL230	6.70	5.89	-0.81 ± 0.92	2.4×10^{-3}	17	paired t-test	17	0.76	1.00	1.00	1.00	Inhibition
phenyl-to-3-pyridinyl	CHEMBL237	8.01	7.40	-0.61 ± 1.01	4.0×10^{-2}	14	paired t-test	15	0.71	1.00	0.93	0.86	Inhibition
phenyl-to-3-pyridinyl	CHEMBL240	5.91	5.39	-0.52 ± 0.52	6.5×10^{-5}	24	paired t-test	25	0.88	1.00	0.96	0.96	Inhibition
phenyl-to-3-pyridinyl	CHEMBL253	7.28	6.62	-0.66 ± 0.74	2.0×10^{-2}	10	paired t-test	11	0.80	1.00	0.90	1.00	Inhibition
phenyl-to-3-pyridinyl	CHEMBL3371	7.95	6.71	-1.24 ± 0.84	1.8×10^{-4}	13	paired t-test	13	0.77	1.00	0.85	0.85	Inhibition
phenyl-to-3-pyridinyl	CHEMBL4766	6.63	7.21	+0.58 ± 0.96	5.1×10^{-6}	92	Wilcoxon Signed-Rank	1	1.00	1.00	1.00	1.00	Inhibition
phenyl-to-4-pyridinyl	CHEMBL230	6.13	5.43	-0.70 ± 1.10	6.1×10^{-2}	11	paired t-test	9	0.73	1.00	1.00	1.00	Inhibition
phenyl-to-4-pyridinyl	CHEMBL236	8.21	7.26	-0.94 ± 0.68	1.1×10^{-2}	10	Wilcoxon Signed-Rank	10	0.40	1.00	0.90	0.80	Inhibition
phenyl-to-4-pyridinyl	CHEMBL3371	7.37	6.70	-0.66 ± 0.79	7.7×10^{-3}	14	paired t-test	9	0.71	1.00	0.93	0.93	Inhibition
ortho-phenylene-to-ortho-phenylene-and-meta-chlorine	CHEMBL214	7.84	7.05	-0.79 ± 0.60	1.1×10^{-5}	20	paired t-test	7	1.00	1.00	1.00	1.00	Inhibition
ortho-phenylene-to-ortho-phenylene-and-meta-chlorine	CHEMBL228	7.51	8.18	+0.67 ± 0.89	8.2×10^{-5}	35	paired t-test	21	0.77	1.00	0.94	0.89	Inhibition
ortho-phenylene-to-ortho-phenylene-and-meta-fluorine	CHEMBL1889	6.75	6.21	-0.54 ± 0.82	4.5×10^{-2}	12	Wilcoxon Signed-Rank	7	0.75	0.83	0.58	0.92	Inhibition

replacement_kind	Target ChEMBL ID	Mean original pChEMBL	Mean replacement pChEMBL	Mean change of pChEMBL ± standard deviation	p-value	Pair count	Test	Unique Documents	Document consistency ratio	Salt form consistency ratio	Assay context consistency ratio	Standard type consistency ratio	Mechanism of action
ortho-phenylene-to-ortho-phenylene-and-meta-fluorine	CHEMBL214	7.73	7.08	-0.65 ± 0.46	2.4×10^{-5}	17	paired t-test	13	0.88	1.00	0.94	1.00	Inhibition
ortho-phenylene-to-ortho-phenylene-and-ortho-fluorine	CHEMBL4722	7.55	8.10	+0.55 ± 0.65	9.9×10^{-3}	13	paired t-test	8	0.92	1.00	0.92	1.00	Inhibition
ortho-phenylene-to-para-phenylene-and-meta-chlorine	CHEMBL228	7.20	8.07	+0.87 ± 0.91	3.6×10^{-3}	14	paired t-test	14	0.86	1.00	0.93	1.00	Inhibition
ortho-phenylene-to-para-phenylene-and-meta-chlorine	CHEMBL279	5.98	6.50	+0.52 ± 0.67	1.7×10^{-2}	13	Wilcoxon Signed-Rank	13	0.46	1.00	0.77	1.00	Inhibition
ortho-phenylene-to-para-phenylene-and-meta-fluorine	CHEMBL222	7.77	7.01	-0.76 ± 0.78	3.0×10^{-3}	14	paired t-test	8	1.00	1.00	1.00	1.00	Inhibition
ortho-phenylene-to-para-phenylene-and-meta-fluorine	CHEMBL230	6.29	6.85	+0.56 ± 0.94	2.1×10^{-2}	13	Wilcoxon Signed-Rank	13	0.46	1.00	0.92	1.00	Inhibition
ortho-phenylene-to-para-phenylene-and-meta-fluorine	CHEMBL4722	7.96	8.54	+0.58 ± 0.82	2.5×10^{-2}	10	Wilcoxon Signed-Rank	4	0.90	1.00	1.00	1.00	Inhibition
ortho-phenylene-to-para-phenylene-and-ortho-chlorine	CHEMBL228	7.20	8.07	+0.87 ± 0.91	3.6×10^{-3}	14	paired t-test	14	0.86	1.00	0.93	1.00	Inhibition
ortho-phenylene-to-para-phenylene-and-ortho-chlorine	CHEMBL279	5.98	6.50	+0.52 ± 0.67	1.7×10^{-2}	13	Wilcoxon Signed-Rank	13	0.46	1.00	0.77	1.00	Inhibition
para-phenylene-to-meta-phenylene-and-ortho-chlorine	CHEMBL4429	7.93	8.51	+0.58 ± 0.48	1.6×10^{-3}	12	paired t-test	3	1.00	1.00	1.00	1.00	Inhibition
para-phenylene-to-para-phenylene-and-ortho-chlorine	CHEMBL1889	7.99	7.42	-0.57 ± 0.95	6.2×10^{-2}	12	paired t-test	6	0.83	0.92	0.83	0.83	Inhibition
para-phenylene-to-2,5-disubstituted-thiophen	CHEMBL3371	7.90	8.44	+0.54 ± 0.36	1.0×10^{-3}	10	paired t-test	6	0.60	1.00	0.60	0.90	Inhibition
meta-phenylene-to-meta-phenylene-and-meta-fluorine	CHEMBL3371	8.77	8.15	-0.62 ± 0.47	1.4×10^{-3}	11	paired t-test	4	0.91	1.00	1.00	1.00	Inhibition

replacement_kind	Target ChEMBL ID	Mean original pChEMBL	Mean replacement pChEMBL	Mean change of pChEMBL ± standard deviation	p-value	Pair count	Test	Unique Documents	Document consistency ratio	Salt form consistency ratio	Assay context consistency ratio	Standard type consistency ratio	Mechanism of action
meta-phenylene-to-meta-phenylene-and-ortho-chlorine	CHEMBL2039	6.11	6.75	+0.64 ± 0.56	1.6×10^{-5}	23	paired t-test	16	0.96	1.00	0.96	1.00	Inhibition
meta-phenylene-to-meta-phenylene-and-ortho-chlorine	CHEMBL228	7.02	7.58	+0.56 ± 0.85	1.4×10^{-3}	29	paired t-test	26	0.79	0.97	0.90	0.97	Inhibition
meta-phenylene-to-meta-phenylene-and-ortho-chlorine	CHEMBL3267	6.57	7.27	+0.70 ± 0.60	3.1×10^{-3}	11	paired t-test	8	0.91	1.00	1.00	1.00	Inhibition
meta-phenylene-to-para-phenylene-and-ortho-chlorine	CHEMBL2039	5.95	6.57	+0.61 ± 0.51	3.0×10^{-7}	30	paired t-test	20	0.93	1.00	0.93	0.97	Inhibition
meta-phenylene-to-para-phenylene-and-ortho-chlorine	CHEMBL221	5.14	5.75	+0.61 ± 0.67	1.8×10^{-2}	10	paired t-test	9	0.70	1.00	0.90	1.00	Inhibition
meta-phenylene-to-para-phenylene-and-ortho-chlorine	CHEMBL228	6.98	7.61	+0.63 ± 0.67	4.3×10^{-6}	34	paired t-test	28	0.77	0.97	0.91	0.97	Inhibition
meta-phenylene-to-para-phenylene-and-ortho-fluorine	CHEMBL203	7.24	6.12	-1.13 ± 1.63	1.2×10^{-2}	17	paired t-test	18	0.65	1.00	0.82	1.00	Inhibition
ester-to-secondary-amide	CHEMBL253	8.83	8.00	-0.83 ± 0.65	9.6×10^{-4}	12	paired t-test	2	1.00	1.00	1.00	1.00	Activation
secondary-amide-to-ester	CHEMBL253	8.00	8.83	+0.83 ± 0.65	9.6×10^{-4}	12	paired t-test	2	1.00	1.00	1.00	1.00	Activation
phenyl-to-phenyl-and-meta-chlorine	CHEMBL213	6.69	7.35	+0.66 ± 1.19	1.5×10^{-2}	12	Wilcoxon Signed-Rank	7	0.92	0.92	1.00	1.00	Activation
meta-phenylene-to-meta-phenylene-and-ortho-fluorine	CHEMBL208	8.50	7.96	-0.54 ± 0.50	3.4×10^{-3}	12	paired t-test	7	0.83	1.00	1.00	1.00	Activation
COOH-to-primary-amide	CHEMBL237	6.21	7.13	+0.92 ± 0.85	5.1×10^{-3}	11	paired t-test	3	1.00	1.00	1.00	1.00	Activation

Table S2. Summary of compound pairs showing pronounced potency shifts at off-target proteins with only minor changes at another known target.

Replacement	Shift target	Shift target mean change	Non-shift target	Mean original pChEMBL at non-shift target	Mean replacement pChEMBL at non-shift target	Pair count at non-shift target	Non-shift target mean change ± standard deviation	Mechanism of action
phenyl-to-cyclohexyl	CHEMBL251	-0.86	CHEMBL226	6.93	6.73	11	-0.21 ± 0.82	Inhibition
phenyl-to-furanyl	CHEMBL251	+0.58	CHEMBL226	7.11	7.25	66	+0.14 ± 0.52	Inhibition
phenyl-to-phenyl-and-meta-chlorine	CHEMBL203	+0.56	CHEMBL279	6.25	6.05	6	-0.20 ± 0.45	Inhibition
phenyl-to-phenyl-and-para-chlorine	CHEMBL240	+0.66	CHEMBL217	6.68	6.83	5	+0.15 ± 0.29	Inhibition
phenyl-to-phenyl-and-para-chlorine	CHEMBL3371	-0.52	CHEMBL214	6.63	6.61	7	-0.02 ± 0.60	Inhibition
phenyl-to-3-pyridinyl	CHEMBL237	-0.61	CHEMBL233	7.79	7.98	12	+0.19 ± 0.83	Inhibition
phenyl-to-3-pyridinyl	CHEMBL240	-0.52	CHEMBL4015	7.70	7.57	5	-0.13 ± 0.50	Inhibition
ortho-phenylene-to-ortho-phenylene-and-meta-chlorine	CHEMBL214	-0.79	CHEMBL229	8.44	8.51	6	+0.07 ± 0.47	Inhibition
ortho-phenylene-to-ortho-phenylene-and-meta-chlorine	CHEMBL228	+0.67	CHEMBL222	7.72	7.70	22	-0.02 ± 0.92	Inhibition
ortho-phenylene-to-ortho-phenylene-and-meta-chlorine	CHEMBL228	+0.67	CHEMBL238	6.21	6.27	15	+0.06 ± 0.59	Inhibition
ortho-phenylene-to-ortho-phenylene-and-meta-fluorine	CHEMBL1889	-0.54	CHEMBL1790	8.36	8.12	6	-0.25 ± 0.54	Inhibition
ortho-phenylene-to-ortho-phenylene-and-meta-fluorine	CHEMBL1889	-0.54	CHEMBL2049	7.51	7.64	6	+0.14 ± 0.30	Inhibition
ortho-phenylene-to-para-phenylene-and-meta-fluorine	CHEMBL222	-0.76	CHEMBL238	6.04	6.20	10	+0.16 ± 0.46	Inhibition
para-phenylene-to-para-phenylene-and-ortho-chlorine	CHEMBL1889	-0.57	CHEMBL1790	7.98	8.05	9	+0.07 ± 0.51	Inhibition
meta-phenylene-to-para-phenylene-and-ortho-chlorine	CHEMBL2039	+0.61	CHEMBL1951	5.79	5.77	12	-0.01 ± 1.06	Inhibition
meta-phenylene-to-para-phenylene-and-ortho-fluorine	CHEMBL203	-1.13	CHEMBL279	5.79	5.88	6	+0.09 ± 0.71	Inhibition
